# Tai Chi Interventions for Slowing Cognitive Decline in Mild Cognitive Impairment: A Review of Randomized Controlled Trials

**DOI:** 10.7759/cureus.103588

**Published:** 2026-02-14

**Authors:** Graham A Branscom, Bianca Vama, Bintou Bane, McKenna Sun, Jianghong Liu

**Affiliations:** 1 School of Arts and Sciences, University of Pennsylvania, Philadelphia, USA; 2 Family and Community Health, University of Pennsylvania, Philadelphia, USA

**Keywords:** aging, cognitive decline, complementary, mild cognitive impairment, review, tai chi

## Abstract

The objective of this review was to compile randomized controlled trials that examined the effectiveness of tai chi in slowing cognitive decline among individuals with mild cognitive impairment (MCI). We limited our review to studies with interventions that lasted at least six months (≥ 24 weeks).

Three databases were searched using similar search terms and inclusion/exclusion criteria to identify studies for review. In total, eight randomized controlled trials were included. The inclusion criteria limited the selected articles to RCTs whose patients all had MCI and were at risk for developing cognitive decline.

All eight studies revealed that tai chi slows cognitive decline among patients with MCI across various domains, including memory, attention, and global cognition, compared to the control groups.

Notably, improvements in patient cognition were consistently observed across studies. Three studies using the Montreal Cognitive Assessment revealed a greater increase in MoCA scores from baseline to follow-up (≥24 weeks) among the tai chi intervention group compared to the non-tai chi control group (0.84, 1.3, and 2.9). Similarly, two studies using the Mini-Mental State Examination found greater cognitive improvements among the tai chi group than in the control group from baseline to follow-up (0.60 and 0.48).

In conclusion, this review supports tai chi as a promising intervention for slowing cognitive decline in individuals with MCI. Positive effects were observed across various cognitive assessments. Thus, these findings may help guide patient care strategies and inform clinical models for MCI management.

## Introduction and background

As accessibility to medical care continues to improve globally, longer life expectancies continue to be observed. By 2030, 1 in 6 individuals will be 60 years or older, and the global elderly population is expected to double from 1 billion in 2030 to 2.1 billion in 2050 [1]. Mild cognitive impairment (MCI) refers to impaired cognition without impairment of social and daily activities, and it is mostly observed among the aging population. An estimated 15.6% of the global population of community-dwelling adults aged 50 years and older have MCI [2]. MCI has been described to affect one or more cognitive domains in learning and memory, language, complex attention, executive function, social cognition, and visuospatial function. The histopathology is heterogeneous, with some studies reporting MCI being associated with the buildup of amyloid plaques (identified via the Bielschowsky silver method) and others noting neuronal loss [3]. Since many presentations of MCI develop into dementia [4], prevention efforts are increasingly important in mitigating the incidence of dementia in elderly populations.

Tai chi, also known as tai chi chuan/quan or taiji, was developed in China by martial artist Chen Wangting at the end of the Ming dynasty (17th century) [5]. Tai chi is a mind-body exercise that incorporates physical, cognitive, and psychosocial components [6]. While the practice of tai chi is extremely popular in the East and is even cited as the most common regular form of exercise in Shanghai, China, it remains an emerging activity in the West whose popularity is gradually increasing [7-9]. From 2007 to 2017, a 64% increase in tai chi engagement was observed among individuals in the United States who participated in the National Health Interview Survey [10]. Tai chi is a highly recommended exercise for older adults due to its low-intensity movements that promote participant safety and its benefits for balance, muscle flexibility, and muscle endurance [11]. Current clinical guidelines recommend that patients with MCI conduct both physical and mental exercise routines to help mitigate further cognitive decline, with some studies investigating tai chi’s use in MCI specifically [12-13]. Learning choreography for tai chi enhances visuospatial processing, processing speed, and episodic memory [14]. Additionally, the sustained attention and multitasking behaviors required for tai chi improve executive functioning while the meditative components reduce susceptibility to anxiety and depression [15].

The aim of conducting this review is to compile available research that meets rigorous criteria and assess the efficacy of tai chi as an intervention for the longitudinal prevention of cognitive decline and dementia. While there are existing review papers on the effects of tai chi on MCI, they are limited in size [16], are not specific to MCI and include other types of cognitive deficits [17], and/or are not exclusively examining long-term interventions and follow-up periods (≥24 weeks) [18-19]. This review adds new knowledge to the literature because of our rigorously defined inclusion criteria, most notably limiting included studies to RCTs whose patients all had MCI and were at risk for developing cognitive decline, and those intervention periods lasting at least six months (≥24 weeks). Since individuals with MCI are at risk of cognitive decline throughout the rest of their lives [3], it is necessary to focus exclusively on longitudinal effects. Thus, this review offers novel insight that can inform researchers and clinicians involved with treating patients with MCI about the role of complementary therapies like tai chi.

## Review

Methods

Search Strategy

**Figure 1:**
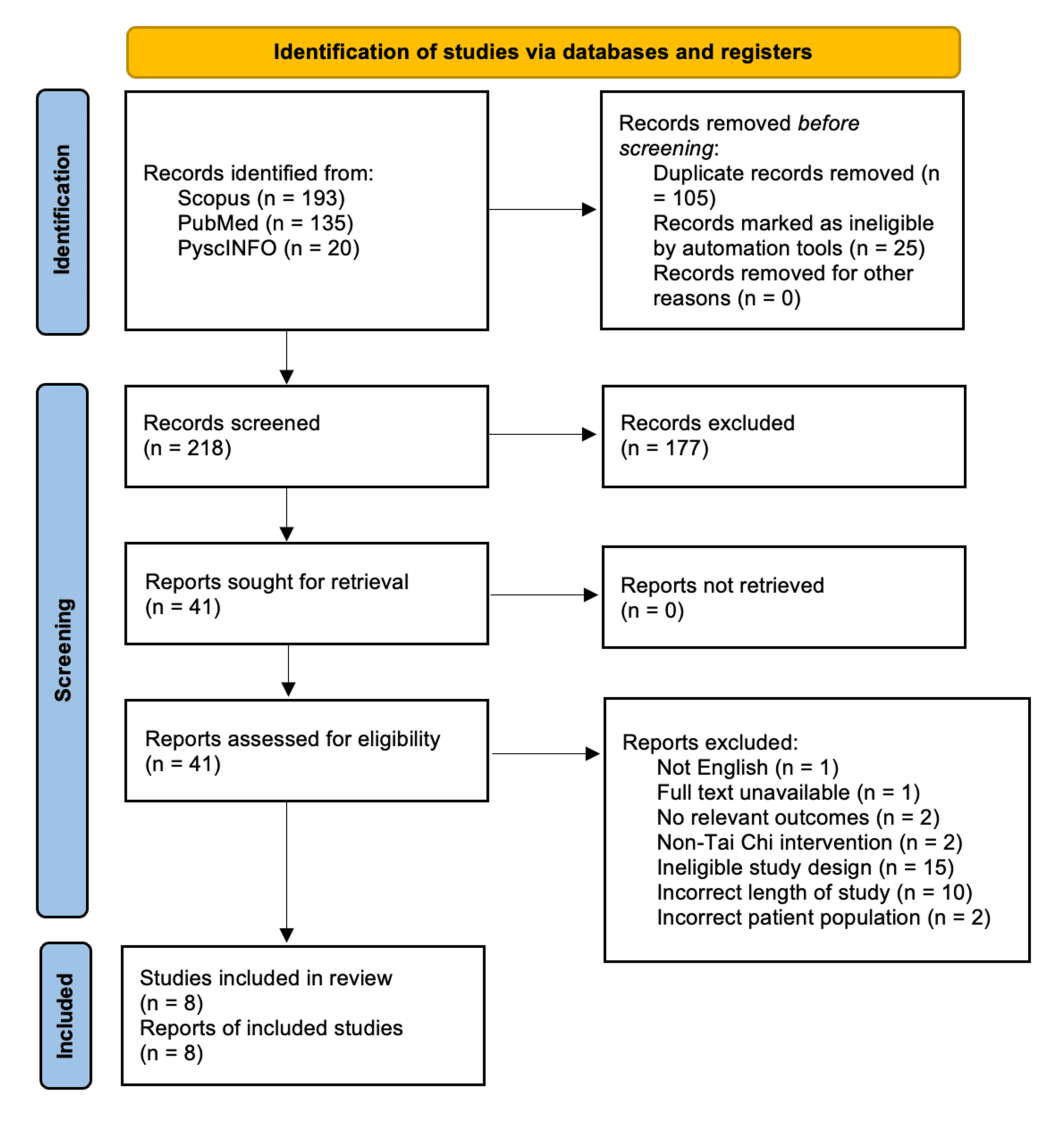
Identifying and screening articles to include in our review Our screening followed the PRISMA guidelines. PRISMA, Preferred Reporting Items for Systematic Reviews and Meta-Analyses

Covidence was used to compile the results from the searches across three separate databases. From a total of 348 studies, an automated screening was conducted to reduce the number of studies to n=218 (Figure 1).

The search strategy we conducted was in consultation with a research librarian in our field. We conducted a comprehensive search across three databases: PubMed, Scopus, and APA PsycINFO (Table 1). We automatically filtered only for papers written in the English language, along with an additional filter for middle-aged patient populations. We then found search terms using the National Library of Medicine’s Medical Subject Headings (MeSH) online tool.

**Table 1:** Search strategy

Database	Search String
PubMed	("Tai Ji"[Mesh] OR "tai chi" OR "chen style" OR "yang style" OR "wu style" OR "sun style" OR "hao style" OR "Acupressure"[Mesh] OR acupressure) AND ("Cognitive Dysfunction"[Mesh] OR "cognitive decline" OR "cognitive dysfunction" OR "cognitive impairment" OR "mild cognitive impairment" OR "Cognition"[Mesh] OR "cognitive function" OR cognition OR "Self Care"[Mesh] OR "self care" ) AND (english[Filter]) AND (middleaged[Filter] OR aged[Filter])
Scopus	("Tai Ji" OR "tai chi" OR "tai ji" OR "Acupressure" OR acupressure) AND ("Cognitive Dysfunction" OR "cognitive decline" OR "cognitive dysfunction" OR "cognitive impairment" OR "Cognition" OR "cognitive function" OR cognition OR "Self Care" OR "self care") AND (“English”) AND (“Middle Aged” or Aged)
PsycInfo	(qigong OR acupressure OR "tai chi" OR "tai j") AND (MAINSUBJECT.EXACT ("Cognition") OR MAINSUBJECT.EXACT("Cognitive Dissonance") _ APA OR MAINSUBJECT.EXACT("Cognitive Ability") OR MAINSUBJECT.EXACT("Cognitive Discrimination" OR MAINSUBJECT.EXACT("Cognitive PsycInfo® Aging") OR MAINSUBJECT.EXACT.EXPLODE("Cognitive Ability") OR MAINSUBJECT.EXACT.EXPLODE(Cognitive Impairment") OR cognit* OR MAINSUBJECT.EXACT("Self-Care Skills") OR MAINSUBJECT.EXACT("Self-Care") OR "self care")

The automated screening included the removal of duplicates or papers deemed otherwise ineligible (non-RCTs). The remaining studies (n=41) were then manually screened to reduce the total number of studies to eight, which composed our final group of studies for review. Screening was conducted based on the following established inclusion criteria.

Inclusion Criteria

Selected papers were limited to studies that were conducted as randomized controlled trials (RCTs) (Table 1). This entailed partitioning participants into an intervention group that completed some form of tai chi exercise and a control group that did not complete the tai chi intervention. Additionally, included studies were limited to those that had intervention periods that lasted at least six months (≥24 weeks) so that the mid- to long-term effects of tai chi could be examined. Although selected studies were not required to include several follow-up times for testing, that was an additional positive characteristic of many of the studies included in the screen. Included studies were also limited to those in which the results were published within the previous 15 years to ensure relevance to the field. The geographical area of research was not limited; included studies were conducted in a broad range of areas around the world, such as mainland China, Taiwan, Hong Kong, Thailand, and the United States. Additionally, studies were limited to patients with certain characteristics. Subjects were all older adults (typically aged ≥ 65 years old), many of whom came from nursing homes. Since this review is interested in the effects tai chi has on preventing cognitive decline in patients who already have MCI, the studied patient population was limited to patients with diagnosed MCI.

**Table 2:** Study characteristics Abbreviations: RCT, randomized controlled trial; wk/wks, week/weeks; BL, baseline; T2D, type 2 diabetes; MCI, mild cognitive impairment; MoCA, Montreal Cognitive Assessment; CCT, computerized cognitive training; MDRS, Mattis Dementia Rating Scale; TICS-M, modified Telephone Interview of Cognitive Status; TUG, timed up and go; ADLs, activities of daily living; ABC, activities-specific balance confidence; TC, tai chi; EX, exercise; CON, control; MoCA-HK, Hong Kong Montreal Cognitive Assessment; CDR, Clinical Dementia Rating; DAD, disability assessment for dementia; CSDD, Cornell Scale for Depression in Dementia; NPI, Neuropsychiatric Inventory; BBS, Berg Balance Scale; AD, Alzheimer’s disease; a-MCI, amnestic mild cognitive impairment; MMSE, Mini-Mental State Examination; LM, Logical Memory; TMT B-A, Trail Making Test Part B minus Part A; BDNF, brain-derived neurotrophic factor; TNF alpha, tumor necrosis factor alpha; IL-10, interleukin 10; CT, cognitive training; MixT, mixed tai chi and cognitive training; AVLT, Auditory Verbal Learning Test; CFT, Complex Figure Test; SCWT, Stroop Color-Word Test; STT-A/B, Shape Trail Test Parts A and B; BNT, Boston Naming Test; ADAS-Cog, Alzheimer’s Disease Assessment Scale–Cognitive Subscale; fMRI, functional magnetic resonance imaging; ALFF, amplitude of low-frequency fluctuation; MTCC, mindfulness-based tai chi chuan; SPPB, short physical performance battery; CDR-SB, Clinical Dementia Rating–Sum of Boxes; DSB, Digit Span Backward; SD, standard deviation

Paper	Study type	Study location	Groups	Interventional timeline	Data collection times (wks)	Study population characteristics	Outcomes tests/organization
Chen et al. 2023 [20]	RCT	Beijing, Fuzhou, Harbin, Shenzhen (China)	Group 1: Tai chi chuan; Group 2: Fitness walking; Group 3: Control	3 times/wk for 24 wks (12 additional wks encouraged training)	BL, 24, 36 wks	N = 328; Age ≥ 60 years; patients with a clinical diagnosis of T2D and MCI; diabetes covariate	Primary outcome: Global cognitive function at 36 wks measured by MoCA. Secondary outcomes: MoCA at 24 wks, other cognitive measures at 24 and 36 wks, blood metabolic indices at 24 and 36 wks
Hwang et al. 2023 [21]	Single-blind randomized trial	Outpatient clinics of Taipei Medical University Hospital, Taipei (Taiwan)	Group 1: CCT + tai chi control; Group 2: Health education interventions	1 time/wk for 24 wks	BL, 26, 52 wks	N=189; Age ≥ 65 years; patients diagnosed with MCI	Primary outcome: Cognitive function measured at 24 and 52 wks by the MDRS and the TICS-M. Secondary outcomes (all at 24 and 52 wks): TUG, Tinetti’s balance, ADLs, ABC
Yu et al. 2022 [22]	Parallel group, assessor-blinded, pilot RCT	From a single research site in Hong Kong	Group 1: Tai chi (TC, n= 10, 3 sessions of 60-min yang-style tai chi training per week for 24 wks), Group 2: Conventional exercise (EX: n= 12, 3 sessions of 60-min fitness training per week for 24 weeks), Group 3: Control (CON: n= 12, no intervention)	3 times/wk for 24 wks	BL, 12, 24 wks	N=34; Age ≥ 50 years; patients with MCI	Primary outcome: MoCA-HK at 12 and 24 wks
Lam et al. 2014 [23]	RCT	Elderly residential homes in Hong Kong	Group 1: “24-style” tai chi; Group 2: Control stretching and relaxation exercises	Phase 1 (induction): 1 time/wk for 4-6 wks; Phase 2 (maintenance): 3 times/wk for the rest (up to week 52)	BL, 8, 26, 52 wks	N=389; Age: >65 years; patients with MCI (CDR of 0.5) or amnestic MCI	Primary Outcome: Rate of conversion to clinical dementia (CDR of 1) after 1 year of intervention and functioning changes as measured by DAD. Secondary outcomes: Change in CSDD score at 1 year, change in NPI (emotional test examines depression, apathy, and anxiety) score at 1 year, change in BBS
Sungkarat et al. 2018 [24]	Prospective interventional RCT	Thailand	Group 1: Tai chi (n=33-4 drop outs); Group 2: Control Educational materials (booklet, 1-hour presentation + q&a discussion section) on cognitive health (n=33-6 drop outs)	Group 1: 50 min session 3 times/week for 6 months; Group 2: 1 hour presentation of cognitive impairment + question discussion section and weekly encouragement of discussion during phone call	BL, 104 wks	N=66; Age: mean age 68; older adults with amnestic MCI, because it is a common and prodromal symptom of AD. Qualified if met Petersen's criteria for diagnosing amnestic multiple-domain MCI (a-MCI), had a score of ≥24 on the MMSE and <26 on the MoCA, had adequate memory with cues, and comprehended instructions.	Primary outcome: Cognitive performance on LM delayed recall, Block Design, Digit Span, and TMT B-A, which examines different cognitive domains. Secondary outcome: Plasma biomarkers including BDNF, TNF alpha, IL-10
Li B et al. 2023 [25]	Prospective, single-blind RCT	Located in RuiJin Hospital, Shanghai Jiao Tong University School of Medicine, Shanghai, China.	Group 1: Online 20- to 30-min CT; Group 2a: MixT, CT, and supervised group 24-form Yang-style tai chi; Group 2b: MixT cognitive training and supervised group 24-form Yang-style tai chi; Group 3: Control participants provided with general health advice	Group 1: 3-4 times/wk for 52 wks; Group 2a: 3 times/wk for 52 wks; Group 2b: 3 times/wk for 104 wks	BL, 26, 78, 104 wks	N= 152 (CT group: N=51, Control: N=53, MixT 1: N=22, MixT 2: N=19); Age (avg.) = 65. Patients with MCI from memory clinics with cognitive complaints for at least 1 year	Cognitive test outcomes: MMSE (26, 52, 104 wks), 5-min and 10-min AVLT, Huashan version (52, 104 wks each), Res-Osterrieth CFT (52 wks), SCWT, STT-A/B (52 wks), BNT (52 wks), ADAS-Cog (26, 52, 104 wks). Imaging outcome: fMRI neuroimaging (voxel-wise mean ALFF), 52 wks
Jiayuan et al. 2021 [26]	RCT	3 communities in Daqing (China)	Group 1: Mindfulness intervention; Group 2: Tai chi chuan intervention; Group 3: MTCC intervention	2 times/wk for 24 wks (two 12-wk stages)	BL, 26, 208 wks	N=93; Age ≥65 years; patients with MCI, confirmed by score of 0.5 on CDR	Primary outcomes: Cognitive frailty measured by Fried Frailty Criteria + CDR. Secondary outcomes: Cognitive function measured by MMSE, physical level measured by SPPB, TUG, 30-second chair test
Li F et al. 2023 [27]	RCT	Community residential homes in the United States	Group 1: Cognitively enhanced tai ji quan (n = 105); Group 2: Standard tai ji quan (n = 107); Group 3: Control stretching (n = 106)	1 time/wk for 24 wks	BL, 16, 24, 48 wks	N = 318; Age: >65; older adults with self-reported memory decline or concern and a CDR global score of 0.5 or lower at baseline	Primary outcome: Global cognition measured by MoCA (range, 0 to 30), dual-task walking costs (difference between single- and dual-task gait speed, expressed in percentage) from baseline to 24 wks. Secondary outcomes: CDR-SB, TMT B, DSB, Physical performance tests

All included studies used assessments for cognitive function that are common in the field of cognitive research. Among the various tests that the studies used, the most common were the Montreal Cognitive Assessment (MoCA), the Trail Making Test (TMT), the Digit Span (DGS) test, the Mini-Mental State Examination (MMSE), and the Alzheimer’s Disease Assessment Scale (ADAS). All these tests are used to assess MCI in patients by taking patients through a series of cognitive tasks of varying difficulty.

Exclusion Criteria

Additionally, by reviewing the papers from the initial search, common characteristics were identified among these studies that did not fit the goals of our investigation. This allowed us to identify several exclusion criteria. We excluded studies that were RCT protocol papers, i.e., only described a study’s protocol without any results, since the trial is still in progress. Also, we excluded studies that were RCT pilot studies, i.e., low-powered studies that served to test out principles before launching a full-scale RCT. We excluded studies that focused on outcomes that were limited to fall risk and insomnia. Lastly, we excluded studies that focused on lessening the symptomatic burden of chronic diseases like cancer, with no primary focus on MCI.

PRISMA

This review followed the Preferred Reporting Items for Systematic Reviews and Meta-Analyses (PRISMA) guidelines. We conducted a risk of bias assessment using the Cochrane RoB 2 tool and generated the risk of bias figure using the “robvis” tool.

Results

**Figure 2:**
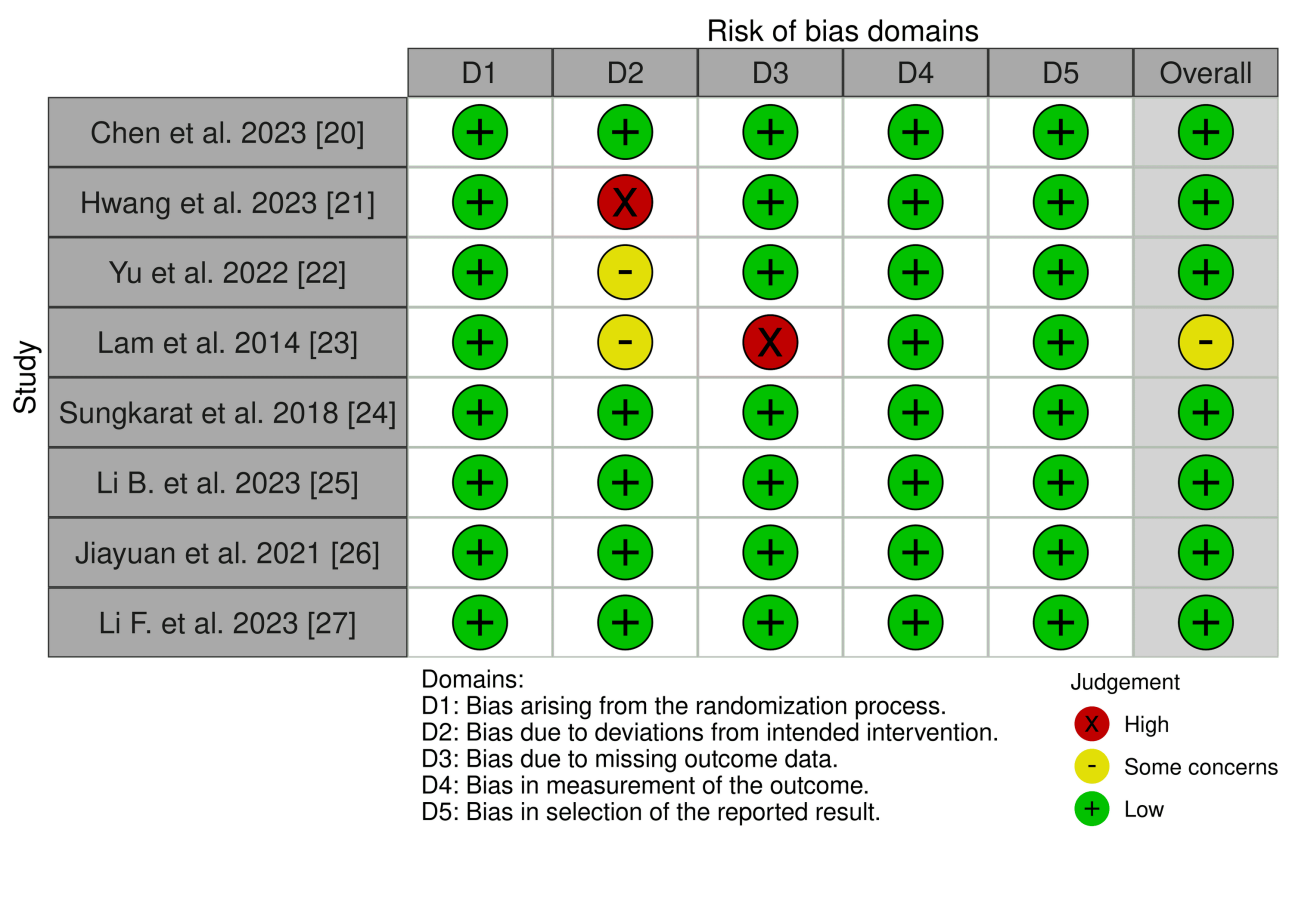
Risk of bias assessment The risk of bias assessment was conducted using the Cochrane Risk of Bias 2 tool.

We determined that 7/8 of our studies had a low risk of bias, and one study (Lam et al. [23]) had some concerns for a risk of bias (Figure 2). Overall, these results suggest that our studies are largely high-quality RCTs.

The synthesized findings from these studies reveal consistent trends of improved cognitive outcomes from tai chi among older adults with MCI, with some variability in terms of the degree of improvement. All studies demonstrated that interventions consisting of tai chi had more beneficial outcomes than the control group(s) engaged in alternative interventions such as walking and health education (Table 2). In five studies [20-24], the tai chi group had higher scores across the various cognitive tests than the control group. The other studies included multiple treatment groups but still demonstrated the highest cognitive performance in the tai chi group ([25-27]). For instance, Li B et al. [25] showed that the group that completed tai chi paired with cognitive training outperformed the groups who engaged in cognitive training alone and the control group.

**Table 3:** Study results and analysis MoCA, Montreal Cognitive Assessment; T2D, type 2 diabetes; wk, week; TICS-M, modified Telephone Interview of Cognitive Status; TUG, timed up and go; ABC, activities-specific balance confidence; ADLs, activities of daily living; CCT, computerized cognitive training; TCE, tai chi exercise; MCI, mild cognitive impairment; MoCA-HK, Hong Kong Montreal Cognitive Assessment; TC, tai chi; EX, exercise; LM, Logical Memory; TMT B-A, Trail Making Test Part B minus Part A; BDNF, brain-derived neurotrophic factor; MMSE, Mini-Mental State Examination; CFT, Complex Figure Test; AVLT, Auditory Verbal Learning Test; BNT, Boston Naming Test; MixT, mixed tai chi and cognitive training; CT, cognitive training; fMRI, functional magnetic resonance imaging; AD, Alzheimer’s disease; PET, positron emission tomography; Aβ, amyloid beta; CF, cognitive frailty; MTCC, mindfulness-based tai chi chuan; SD, standard deviation.

Paper	Results	Limitations	Adherence data
Chen et al. 2023 [20]	MoCA score: tai chi (24.67) > walking (23.84); 37 adverse events	T2D comorbidity reduces generalizability; 36 wk follow-up period; self-reported calories and physical activity; need objective measures; non-blind participants and assessors —> bias in cognitive assessments	Strong adherence and low loss to follow-up due to highly supervised intervention; No mention of specific efforts to increase adherence; Specific attendance statistics: 88.8% of the tai chi group and 90.0% of the fitness walking group attended more than 75% of planned interventions (54+ sessions), Mean number of sessions completed: 63
Hwang et al. 2023 [21]	Tai chi increased scores on TICS-M + TUG + Tinetti’s balance + ABC + ADLs; small but long-lasting improvement	High attrition rates in CCT and TCE groups. The study did not employ virtual adherence strategies for this study. Survivor effect: cognitive outcomes overestimated in subjects who completed trial home-based administration; cannot ensure adherence during self-training. No follow-ups for study groups. Unknown whether sig. effects in cognitive and secondary outcomes were clinically meaningful for older MCI adults. Behavioral changes in physical and mental activity were not measured —> cannot determine if cognitive improvements come from educational intervention	Both the CCT and TCE groups exhibited high attrition rates; No details on adherence efforts; Provided reasons for high attrition: Low previous experience with computers, intervention times too long, not enough training time to adjust to the intervention, Indirect targeting of cognitive domains not stimulating or easy to conceptualize, People don't see the direct connection of the intervention to specific skills
Yu et al. 2022 [22]	Both exercise groups' improvement: MoCA-HK at 12, 24 weeks. Only tai chi improvement: MoCA-HK at 12 weeks, TMT B/A ratio improvement at 12, 24 weeks. Other tests: Both regular exercise and tai chi showed improvements	Social interaction with instructor and fellow participants could have improved cognitive function; intervention venue provided environmental stimulation for exp. group —> greater cognitive improvement, though this does not affect differences from start to end between groups. Left out two cognitive domains: perceptual-motor function and social cognition. Larger sample size needed to confirm differences in long-term benefits between 2 exercise modalities	No mention of efforts to promote adherence or participation; reported attendance rates: TC: 80.6%, Conventional EX: 77.5%
Lam et al. 2014 [23]	Tai chi group had a lower chance/odds ratio of developing dementia at 52 weeks; similar improvements across BOTH groups in digit/visual backward spans, delayed recall, category verbal fluency test, Alzheimer’s Disease Assessment Scale	Intervention group had higher levels of education; control for differences in educational attainment before interpreting group differences in cognitive test scores. High dropout rate in intervention group —> need preventative measures to boost adherence in high-intensity programs; small sample size limited statistical power in detecting differences in cognitive function between groups; one-year observation period; no long-term benefits of exercise observed	Percentage of subjects who completed assessments: At 1 year: 92 (53.8%) of the intervention group, 169 (77.5%) of the control group; specific efforts to improve adherence: Tai chi masters provided refresher lessons every month. Exercise center logged attendance to assess adherence; higher dropout rate in the intervention group, which had more highly educated subjects.
Sungkarat et al. 2018 [24]	LM test, TMT B-A test, and plasma BDNF were all improved in the tai chi group compared to the control group	The tai chi and control groups were not attention-matched; tai chi group had more interaction with research staff, potentially influencing cognitive function and BDNF levels. All participants were instructed by the same instructor, limiting generalizability to settings with multiple instructors. The small sample size, not determined for plasma data, may lack sufficient power to evaluate tai chi's effects on plasma outcomes. A larger randomized controlled trial with an attention-matched control group and long-term follow-up is needed to confirm these findings. The required "dose" (intensity, duration) of tai chi training for effects on circulating inflammatory cytokines is yet to be determined. 10 participants withdrew	Adherence-promoting strategies: Family members reminded participants, posted notes about exercise schedule as visible reminders, weekly reminder telephone calls, weekly phone calls from research staff. Monitored health and routine changes (e.g., recreation club attendance, supplement and medication use, illnesses, hospital admissions). Intervention participants reported frequency and duration of tai chi exercise and any adverse events. Dropout rate: 10 participants; attendance recorded: 83.6%
Li B et al. 2023 [25]	MMSE, CFT, AVLT, BNT: MixT > CT > Control; fMRI: MixT and CT have increased regional activity —> tai chi enhances CT improvement	Need sample size of 200 per group to provide statistical power; AD diagnosis was confirmed by PET for AD-specific biomarker Aβ; not performed in all MCI patients at baseline	Telephone interviews were used to ensure protocol compliance. No mention of interview frequency. Flowchart includes participants who missed interventions or follow-ups; Cited reasons for higher compliance: Interpersonal characteristics of the MixT intervention, cited higher dropout likelihood for patients with MCI without medications who may opt for medical interventions; specific attendance during interventions not mentioned
Jiayuan et al. 2021 [26]	Lowest level of CF in MTCC group; MTCC group had highest improvement in MMSE; MMSE showed significant Group x Time interaction	Did not use validated biochemical measures for CF older adults to investigate intervention mechanisms. Multicenter communities were in one province, which reduces generalizability; no clear control group (mindfulness is not really a negative control)	All participants completed the interventions; loss to follow-up rate was very low (2.75% = 2 participants). No mention of methods or reasoning behind the high participation rate; no attendance statistics provided.
Li F et al. 2023 [27]	MoCA and dual-task walking: Cognitively enhanced tai chi > standard tai chi and stretching	Test administered online. No non-exercise control group; could not measure net gains in experimental variables. Patient population highly educated and cognitively high-functioning; limits generalizability	Protocol-adherent course of intervention was considered ≥75% of attendance; adherence rate: average 6-month adherence rate was 80% in all groups; attrition rate: 10%, with 33 dropouts. The median dropout time: 8 weeks, (range: 2 to 20 weeks); mean number of completed sessions: overall: 38 sessions (SD 9.7), cognitively enhanced tai ji quan: 38.7 sessions (SD 10.1), standard tai ji quan: 38.2 sessions (SD 10.2), stretching: 38.3 sessions (SD 9.1), interventionists' adherence (fidelity): mean 97% (range: 94% to 98%)

The types of cognitive tests varied across the different studies, demonstrating that the improvement among the tai chi groups was consistent across different cognitive domains. Chen et al. [20], Yu et al. [22], and Li F et al. [27] used the MoCA to examine mental flexibility and frontal lobe function through tasks like the clock drawing test. These three studies revealed that the tai chi intervention group had a greater increase in MoCA scores from baseline to follow-up (≥24 weeks) compared to the non-tai chi control group by 0.84, 1.3, and 2.9 points among Chen et al. [20], Yu et al. [22], and Li F et al. [27], respectively. Jiayuan et al. [26] and Lam et al. [23] utilized the MMSE, which emphasizes memory and language function. These two studies observed improvements among the tai chi group compared to the control group from baseline to follow-up (0.60 and 0.48 in Jiayuan et al. [26] and Lam et al. [23], respectively).

Jiayuan et al. [26], Lam et al. [23], and Li F et al. [27] all utilized the Clinical Dementia Rating (CDR). Unlike the MoCA and MMSE, which use a predetermined set of questions, the CDR is performed through a less structured interview format. Jiayuan et al. [26] revealed that cognitive frailty, as measured by the CDR, exhibited a 12.9% reversal rate among the tai chi group compared to 6.7% among the control group. Lam et al. [23] showed a statistically significant difference in CDR scores in the tai chi vs control group (p=0.038). The tai chi group had a 0.6 point higher improvement in CDR scores from baseline to 24 weeks compared to the control group.

The last commonly shared test across multiple studies is the DGS. Three studies [22, 23, 27] all used the DGS, which diversifies the testing medium used to determine cognitive function since the DGS does not rely on questions or interviews but instead flashes numbers to be recalled forward or backward. Yu et al. [22] reported that the tai chi group had a greater increase from baseline to 24 weeks in the number of digits recalled in the forward and backward DGS, as compared to the control group by 1.5 and 0.9 digits, respectively. The same metric was 2.2 and 2.2 digits in the forward and backward DGS, respectively, in Li et al. [27], but was not statistically significant (p>0.05) in Lam et al. [23].

The studies all included longitudinal (≥24-week) follow-up times and varied in terms of the length of the intervention, allowing us to assess how improvements in cognitive impairment changed over time. Most studies conducted the tai chi intervention for 24 weeks, while other studies extended the intervention for a longer period of 52 or 104 weeks [23, 25]. Data collection timelines typically consisted of four measurements: at baseline before treatment, at midway through the treatment period (8-16 weeks after the start of the intervention), at the end of the treatment period (24-104 weeks after the start of the intervention), and finally at several weeks after the treatment period was over (0 weeks to 3.5 years after the end of the intervention). Long-term benefits of tai chi were observed across multiple studies. For instance, Jiayuan et al. [26] saw MMSE scores continue to increase at six months and even three years after the end of the tai chi intervention period, with the highest scores in the tai chi group. Li B et al. [25] observed greater increases in scores in four out of the six cognitive tests for the tai chi group compared to the control at the first 12-month follow-up period. However, Lam et al. [23] did not show a statistically significant difference (p>0.05) between the tai chi and control groups at one year in any test except for the CDR sum of boxes (p=0.038).

In addition to cognitive parameters, several studies demonstrated improvements in overall mental well-being, such as decreases in depression rates [22, 26]. Tai chi may have a broader effect beyond cognitive abilities.

In summary, the synthesized evidence from these studies suggests that tai chi has a positive impact on various cognitive domains, including memory, attention, and global cognition. The accessibility and safety of tai chi due to its low-impact nature present tai chi as a promising intervention for preventing cognitive decline in older individuals with MCI.

Discussion

Overview

The studies included in this review have examined the cognitive effects of tai chi interventions that lasted for at least 24 weeks and demonstrated improvement in global cognition, emotional health, memory, and executive functioning of older adults with MCI. Additionally, multiple sources have hypothesized cellular and broader-scale mechanisms by which these benefits are imparted to tai chi participants. A common theme across these studies is the support of tai chi as a cognitive health intervention due to its engagement accessibility, i.e., low-impact nature of tai chi exercise, allowing participation with minimal risk of injury, and its financial accessibility, imparted by the lack of expensive equipment or additional resources. In addition, tai chi can be closely tailored to the MCI population, as demonstrated by the cognitive benefits arising from tai chi’s supplementation with mindfulness training or other enhancements such as computerized cognitive training [21, 25-27]. Supporting the exploration of adaptable interventions like tai chi may provide more targeted treatments for maintaining cognitive health.

Our review demonstrates tai chi interventions as a valuable tool for the prevention of MCI progression to dementia by facilitating cognitive engagement, a key factor in the delay of cognitive decline. Nonetheless, optimal tai chi protocols for cognitive enhancement in MCI populations are yet to be developed, and comparisons of MCI-specific tai chi intervention enhancements have yet to be examined.

Critical Appraisal of Included Studies

The major strengths of the studies included in this review are their experimental design status as RCTs, having longitudinal interventional periods, focusing on older adults with MCI, using a diverse array of cognitive tests, and having large sample sizes (34-289 participants). However, studies were limited by the geographical diversity of the study location, variable adherence rates, and an inability to ensure a consistent home environment.

Due to the personalized nature of traditional Chinese medicine, there has historically been an underrepresentation of evidence-based medicine that investigates the efficacy of traditional Chinese medicine practices [28]. Thus, by focusing strictly on RCTs that blinded assessors and trainers, we ensured that the included studies objectively assessed the impact of tai chi on cognitive function. Li B et al. [25] proposed that informing subjects of the study hypothesis during observation and follow-up enhanced the study’s validity as it reduced the chances for the Hawthorne effect to influence findings. However, it is important to consider that participants’ expectations of intervention benefits may have contributed to a more active lifestyle in excess of the additional physical activity provided by the intervention.

Furthermore, the other component of utilizing RCTs is the presence of at least one control group. Four studies [20, 22, 23, 27] had control groups that were instructed to perform stretches or light physical activity, which allowed researchers to differentiate the effects of tai chi and general non-tai chi physical exercise on cognitive outcomes. Jiayuan et al. [26] had a control group that only received mindfulness meditation training, which allowed researchers to assess the differential effects of tai chi versus mindfulness. Finally, all studies had a health education or non-intervention-related control group except for Li F et al. [27], which only had a “stretching” control group. Including these control groups allowed for a better understanding of the benefits of tai chi and tai chi-related interventions on cognition that derive from the intervention.

An additional strength across these studies was the use of an interventional period of at least 24 weeks and follow-up periods thereafter. Assessing tai chi’s long-term impact on attenuating cognitive decline is particularly important since patients with MCI are often interested in preventing further cognitive deterioration throughout the rest of their lives. By using longitudinal intervention inclusion criteria in our review, we are able to show the long-term improvements from tai chi. All studies showed some increase in cognitive performance in the tai chi group compared to the control at these longitudinal follow-up periods. However, studies like Lam et al. [23] only saw improvements in a small number of the total tests that they measured, demonstrating that tai chi may not cause a unilateral improvement across all cognitive domains.

All reviewed studies focused on patients who are older adults with MCI. By focusing on this specific population, these studies have the most implications for older patients with MCI who want to prevent further cognitive decline. Additionally, all studies - with the exception of Chen et al. [20] - excluded patients with co-occurring mental or physical conditions to improve data validity and reduce the likelihood of confounding variables impacting the assessment of a link between tai chi or tai chi-related interventions and cognitive benefits. It is important to note that Chen et al. [20] demonstrated similar results to the other included studies, adding to the external validity of tai chi and tai chi-related interventions for the general population.

In addition, a wide range of exams was used to assess cognitive functions within and across the included studies. While Yu et al. [22] assessed cognitive function with 10 different cognitive exams and Hwang et al. [21] assessed cognitive function with two cognitive exams, they both assessed at least four different cognitive domains. Although there was not a single study that was individually able to assess all cognitive domains and subdomains, the major cognitive domains of executive function, memory, attention, and language were examined across the studies. All included studies in this review assessed participant cognitive function at baseline and at the end of the intervention. Four studies [20, 23, 25, 26] even conducted follow-up cognitive tests within the time periods ranging from nine weeks to one year post-intervention to determine potential long-term effects of their respective interventions.

Furthermore, five out of the eight studies had large sample sizes of over 100 participants [20, 21, 23, 25, 27]. These large participant cohorts allow for the generalizability of results to the broader population, which in turn strengthens the support for tai chi as a feasible intervention. Conversely, the generalizability of this review is limited as only Chen et al. [20] and Li F et al. [27] recruited participants from diverse communities in their respective countries. The remainder of the included studies either did not specify the exact location where recruitment was conducted or had recruitment limited to their local community in one city. Additionally, despite not excluding any region in our search criteria, all studies were conducted among patients in East and Southeast Asia, except for Li F et al. [27], which was conducted among community residential homes in the US. This geographically limited patient population limits the generalizability of results to other regions, demonstrating the need for further research on tai chi’s cognitive effects among the elderly in different regions across the world.

Another limitation observed included difficulty in maintaining adherence to the interventions. Variable adherence introduces a potential skew in results from survivor bias. Although Chen et al. [20] were able to promote a high adherence rate and low rate of participant loss through extensive follow-up efforts, they cite the high expense of human resources to attain these results, which may not be accessible for the general population and all medical centers. Conversely, Jiayuan et al. [26] reported a high adherence rate to the intervention and the post-intervention follow-ups without detailing extensive human efforts, suggesting a potential difference in population culture. An assessment of the effectiveness of different adherence methods for patients with MCI is a useful endeavor that ought to be conducted before establishing tai chi as a preventative intervention.

Certain confounding variables pose additional limitations on these studies. The most potentially limiting confounding variable was the inability of the studies to control the home environment in which participants were instructed to engage in the experimental intervention, cognitive test, or control group activity [21, 25, 27]. While some studies provided video resources or virtual monitoring with platforms like Zoom for participants to use during the intervention sessions as a method to maintain consistency, participants' ability to mentally and physically participate in the interventions, cognitive tasks, and physical tasks may be impacted by differing home environments, such as noise disturbances. Even with virtual monitoring, however, Hwang et al. [21] mentioned how participants' previous experience with computers may affect their engagement with the intervention, which is especially applicable to the studies that had virtual interventions.

Despite these limitations, the included studies have an abundance of strengths. With the urgent need for a safe preventative intervention for MCI patients and the potential of tai chi to prevent MCI’s decline into dementia, it is important to further research the most effective way of administering tai chi to MCI patients.

Potential Mechanisms

The observed cognitive improvements in older adults with MCI may be attributed to various underlying mechanisms discussed in the studies reviewed. Namely, improved function of specific brain regions and the differential expression of certain neurological biomarkers are the main potential mechanisms.

A common mechanism discussed across the studies is the improved function of different brain regions. Chen et al. [20] posited that the constant memorization and learning of movements in tai chi may be pivotal in achieving better cognitive performance due to the involvement of the prefrontal cortex (PFC), temporal cortex, and hippocampus. Similarly, Jiayuan et al. [26] noted how mindfulness practice in tai chi could enhance functional brain connectivity in the default mode network, potentially reducing hippocampal volume atrophy. The use of fMRI scanning by Li B et al. [25] complemented these results by reporting increased neural activity in brain areas related to working memory and executive functioning, as evidenced by an increased amplitude of low-frequency fluctuations (ALFF) in the middle/superior frontal gyrus and anterior cerebellum lobe of older adults after memory and social support interventions. More generally, Yu et al. [22] and Lam et al. [23] noted how the cognitive benefits of tai chi may be due to broader stimulation across the central nervous system. Additionally, in an fMRI study comparing tai chi and aerobic exercise for eight weeks in 36 college students, Cui et al. found stronger effects on brain plasticity among the tai chi intervention group as evidenced by increases in grey matter volume in specific brain regions and enhanced functional connectivity between the left middle frontal gyrus and left superior parietal lobule. It is hypothesized that tai chi uniquely integrates consciousness, breathing, and movement to enhance synaptic plasticity [29].

Several studies suggest that the modulation of various biomarkers, particularly brain-derived neurotrophic factor (BDNF), contributes to the cognitive benefits of tai chi in individuals with MCI. BDNF is known for its involvement in synaptic plasticity and neurogenesis [30-31]. Multiple studies, including Chen et al. [20] and Li B et al. [25], underscore the role of BDNF in mediating the cognitive benefits of tai chi. Chen et al. [20] proposed that the muscle-strengthening components of tai chi induce the release of BDNF, insulin-like growth factor 1, and homocysteine, all of which contribute to improved cognitive function in the domains of memory and executive functioning. This aligns with the results of a study conducted by Solianik et al. [32], where a 10-week tai chi intervention increased levels of neuroplasticity biomarkers such as BDNF, highlighting potential mechanisms mediating the observed cognitive benefits. Consistent exercise-induced upregulation of BDNF may occur during long-term tai chi interventions (at least 24 weeks) as implemented in the reviewed randomized controlled trials of this paper and may provide a foundation for the observed sustained cognitive improvements [33].

Overall, the mechanisms underlying the cognitive benefits of tai chi in individuals with MCI likely involve both the stimulation and functional preservation of certain brain regions and the modulation of neuroplasticity biomarkers, such as BDNF and LRP1. These findings suggest that tai chi acts as a holistic intervention that combines physical exercise with cognitive engagement, promoting cognitive well-being. Further research is needed to unravel the intricate interplay of these mechanisms and their specific contributions to cognitive improvements in individuals with MCI.

Limitations of This Review

A limitation of this review was that only papers with English versions available were included. Future reviews could improve upon this by using all of our inclusion criteria, but also including papers that can only be found in non-English languages. As tai chi is a Chinese tradition, the Chinese medical literature may offer additional insights that cannot be found in English papers.

*Implications*

Our findings from the synthesis of these studies have important implications for geriatric care. MCI is increasingly prevalent among older adults and often precedes severe cognitive decline and dementia. As the global population ages, finding effective, accessible, and sustainable interventions to maintain cognitive function and prevent cognitive decline is crucial. Our inclusion criteria focused mainly on patients who are older adults, particularly in the 65 years and older range, which provides support for tai chi as a cognitive decline prevention intervention for the elderly population. Furthermore, many studies noted that they were done specifically within elderly residential communities, including Lam et al. [23] and Li F et al. [27], showcasing tai chi's accessibility and adaptability for older adults with different living situations, as it may be practiced in various settings, from residential communities to individual homes. Additionally, since these studies used longitudinal follow-up times, they hold particular importance for older adults who are looking for preventative measures that are focused on long-term decreases in cognitive decline. These studies all showed cognitive improvements across various domains in the tai chi group compared to the control group. Tai chi was associated with improvements in both cognitive tests (e.g., MoCA, CF, MMSE) and clinical markers (e.g., fMRI scans, plasma BDNF). Therefore, physicians who care for elderly patients should be encouraged to recommend tai chi as a demonstrably effective and easily accessible form of exercise. Most patients can easily start doing tai chi at their own homes as soon as they want to. In addition to older patients with MCI, some studies, like Chen et al. [20], note how an early initiation of incorporating tai chi into everyday life is important and can be extrapolated to other populations, such as those with type 2 diabetes, which often coexists with MCI in older adults.

## Conclusions

This review underscores the positive effects of tai chi on preventing cognitive decline in individuals with MCI, as evidenced across all eight studies and a range of cognitive assessments. While these studies highlight several strengths, further research is needed to address key limitations, including diversifying the study population, increasing adherence to the study protocol, and controlling for the standard quality of intervention by ensuring a standardized home environment.

Overall, these findings support the integration of tai chi into clinical and community settings as a viable and accessible intervention for individuals with MCI. They also lay the groundwork for further exploration and refinement of tai chi interventions in diverse populations and settings.
